# T cell-derived small extracellular vesicles in cancer–immune interactions

**DOI:** 10.1007/s00262-025-04109-w

**Published:** 2025-06-25

**Authors:** Ma Janelle Chichoco Garcia, Su Su Thae Hnit, Elena Shklovskaya, Yuling Wang

**Affiliations:** 1 School of Natural Sciences, Faculty of Science and Engineering, Macquarie University, Sydney, NSW 2109 Australia; 2 Macquarie Medical School, Faculty of Medicine, Health and Human Sciences, Macquarie University, Sydney, NSW 2109 Australia

**Keywords:** T cells, Small extracellular vesicles, Intercellular communication, CD4^+^ T cells, CD8^+^ T cells

## Abstract

Small extracellular vesicles (sEVs) are lipid-encapsulated nanoparticles released following the endocytic fusion of multivesicular bodies with the plasma membrane. sEVs are secreted by most eukaryotic cells, and they contain proteins, RNAs and DNA. They act primarily as mediators of intercellular communication through the transport of their contents from donor to recipient cells. Immune cells, including T cells, secrete sEVs following activation. T cell-derived sEVs (T-sEVs) have gained attention in cell-to-cell signalling and as promising immunotherapeutic agents. Growing evidence suggests that T-sEVs are key players in cancer immunotherapy responses. A better understanding of T-sEVs production and properties is key for grasping their biological functions. Extensive current literature on tumour-derived sEVs and their applications in diagnostics or therapeutics is in disconnect with fewer reports on T-sEVs. In this review, we discuss T-sEV biogenesis, their roles in cell-to-cell communication and potential applications in immunotherapy for cancer.

## Introduction

Extracellular vesicles (EVs) are lipid-bound small packages that contain active components secreted from the cell of origin into extracellular space [1]. EVs are classified depending on their size and biogenesis: exosomes (30–150 nm), microvesicles (50–1000 nm) and apoptotic bodies (50–5000 nm) [1–7]. International Society for Extracellular Vesicles (ISEV) categorized EVs into two categories, namely small extracellular vesicles (sEVs) < 200 nm in size and large EVs (> 200 nm) [7–9]. sEVs are lipid bilayer membrane nanosized vesicles [1, 10–13] containing molecules such as tetraspanins, lipid raft-interacting proteins, cytoplasmic proteins, receptors, mRNA, miRNA and other non-coding RNAs [14–17]. sEVs are generated through the intraluminal vesicles (ILVs) from multivesicular bodies (MVBs) of the late endosomes [13, 18–20]. The MVB fusion with the plasma membrane leads to sEVs release [21–24]. sEVs are present in human biological fluids and play a key role in mediating physiological and pathophysiological functions [2, 12, 18, 25, 26].

T cells play a crucial role in adaptive immune responses [27]. T cells generate T cell-derived sEVs (T-sEVs) that act like their parent cells by engaging in antigen-specific interactions, destroying target cells and transporting cytokines [28]. sEVs mediate immune stimulation by carrying signalling molecules and antigens from T cells, and once released, they interact with various immune cells affecting their behaviour [20].

Compared to cell therapy, immune cell-derived sEV-based cancer therapy has shown dynamic potential owing to its ability to cross biological barriers, biocompatibility, low toxicity and non-immunoreactive properties. The immune cell-derived sEVs are altered through loading of specific RNAs or proteins and surface refinement. Though both methods are beneficial, immune cell-derived sEV-based exhibited an edge in cancer therapeutics [29].

There have been many excellent reviews on different cell-derived sEVs for biomarker discovery and therapeutic targets [30, 31]. Despite that, information on the role T-sEVs plays in intercellular communication remains limited. In this review, we discuss the biogenesis and molecular composition of T-sEVs with the focus on intercellular communications via T-sEV-derived microRNAs and cytotoxic proteins.

## T cells and T cell activation

T cells are responsible for cell-mediated adaptive immunity [31, 32]. They carry T cell receptors (TCRs) associated with the CD3 signalling complex [32–35]. TCRs are produced through random V(D)J gene recombination in individual T cells, generating unique TCRs capable of antigen-specific recognition [35]. The T cell membrane contains numerous proteins that participate in activation, proliferation, differentiation and effector functions of T cells [27]. There are two subsets of T cells, CD4^+^ (helper) and CD8^+^ (cytotoxic) [36–40]. CD8^+^ T cells eliminate virus-infected, stressed or malignant cells [29, 37, 41] by secreting lytic granules containing granzymes and perforin [30]. CD4^+^ T cells regulate adaptive immune responses [37, 42–44]. Upon antigen recognition, CD4^+^ T cells differentiate into distinct functional subsets that “help” other immune effector cells ensuring short-term pathogen clearance and long-term immunological memory [26, 36, 45]. In cancer, CD4^+^ T cells may improve immunity by releasing effector cytokines such as interferon-gamma (IFNγ) and tumour necrosis factor alpha (TNFα), and assisting cytotoxic and antibody responses [45, 46]. Alternatively, CD4^+^ T cells which underwent peripheral activation differentiate into induced regulatory T cells [43].

**Fig. 1:**
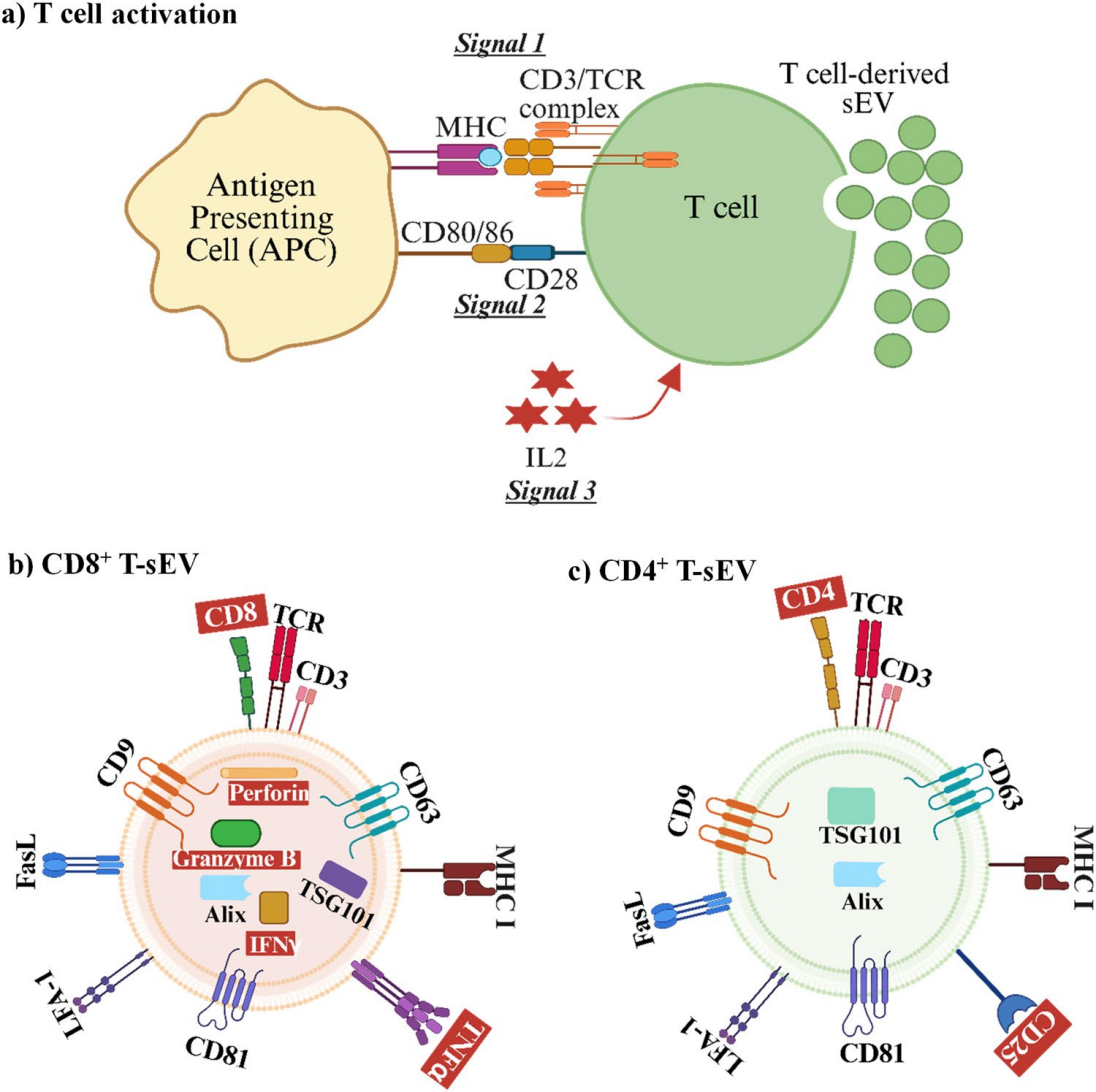
T-sEV generation by T cells activation and specific proteins found on CD8^+^ and CD4^+^ T-sEVs. **a** T cell activation is via *Signal 1* which is provided by the TCR recognition of a peptide antigen bound to the MHC molecule on the APC. The second co-stimulatory signal (*Signal 2*) is provided by CD28 on T cells binding to CD80 and CD86 on APCs. The third signal (*Signal 3*) is T-cell derived IL2. The three signals integrate to stimulate sEV release from the T cell (32). **b** CD8^+^ T-sEVs and **c** CD4^+^ T-sEVs both express the TCR/CD3 complex, MHC I, LFA-1, FasL and the sEV markers Alix, TSG101, CD9, CD63 and CD81. The key differences between CD8^+^ T-sEVs and CD4^+^ T-sEVs are highlighted in a red/dash box. CD8^+^ T-sEVs express TNFα, IFNγ, perforin and granzyme B (85). CD4^+^ T-sEVs express CD25 (116). (Created with BioRender.com)

Three signals are required for T cell activation [47, 48] (Fig. 1a). TCR recognition of peptide antigens presented by either major histocompatibility complex class I (MHC-I) or MHC class II (MHC-II) molecules on antigen-presenting cells (APCs) provides *Signal 1* [49–53]. Notably, both foreign and mutated self-antigens (such as neoantigens in cancer) can generate strong T cell responses. Costimulatory molecules, such as CD80/CD86 on the same APC, provide the second activation signal (*Signal 2*) by engaging with CD28 on T cells [54, 55]. Finally, the third signal (*Signal 3*) is a cytokine such as interleukin2 (IL2) that acts as a T cell growth factor [54, 55]. In cancer, the three signals initiate proliferation and differentiation of CD4^+^ into T helper 1 (Th1) cells while suppressing progression towards other lineages [56], while CD8^+^ T cells differentiate into cytotoxic T cells [57]. T cell activation is marked by expression of MHC-II, CD25 and CD69 [33], and initiates T-sEV release. T cell subset differentiation is associated with expression of new proteins that will allow effector T cells to travel to and enter tissues, and include adhesion molecules such as LFA-1 and CD54, and chemokine receptors such as CXCR3 [33].

## T cell-derived small extracellular vesicles (T-sEVs)

### sEV biogenesis

sEVs originate from endocytosis as intraluminal vesicles (ILVs) following the inward budding of the limiting membrane of multivesicular bodies (MVBs) (Fig. 1b) [57, 58]. ILV functions in the breakdown and suppression of endocytosed receptors through ubiquitination of the receptor cytosolic tail [59, 60]. The early endosome merges with endocytic vesicles, then the contents bound for breakdown, recycling or exocytosis are assimilated into vesicles [26]. Early endosomes are subsequently reorganized leading to late endosome formation or MVBs [26, 61, 62]. MVB maturation is characterized by cargo sorting, followed by fusion with lysosomes for breakdown or with plasma membrane to be secreted as sEVs [63]. One of the key players in endocytic and secretory pathways are soluble NSF (N-ethylmaleimide-sensitive factor) attachment protein (SNAP) receptors (SNAREs), a superfamily of proteins which modulate merging and intracellular vesicle trafficking [64].

The production of sEVs requires the sorting of sEV-targeted proteins and lipids onto the endosomal membrane, the transporting of the sEV cargo into ILVs and the pinching off of ILV [65]. The proteins governed towards ILVs are regulated by a sorting signal such as monoubiquitination of cytosolic domains of transmembrane proteins derived from the trans-Golgi network or the cell surface [66]. This sequential fusion of the MVBs with the plasma membrane resulting in release of sEVs into the extracellular space is regulated by endosomal sorting complexes required for transport (ESCRT) [63]. ESCRT is a group of about 20 proteins which form the complexes, ESCRT-0, -I, -II and ESCRT-III, together with the accessory proteins, ALG-2-interacting protein X (ALIX), vacuolar protein sorting-associated protein (VPS4) and vesicle trafficking 1 (VTA1) [67, 68]. ESCRT-0, -I and -II complexes function in engaging and isolating ubiquitinated membrane proteins found on the endosomal delimiting membrane, while ESCRT-III operates in vesicle scission and membrane budding [67, 68].

For sEVs, the tetraspanins CD9, CD63 and CD81 serve as their surface markers [69, 70]. On the other hand, sEVs contain high levels of lipids such as cholesterol, ceramides and sphingomyelin, which are found in lipid rafts or condensed membrane domains [65]. There are also copious amounts of lipid lysobisphosphatidic acid (LBPA) in the internal membrane of multivesicular endosome (MVE), and they are responsible for the back fusion of ILV with the MVE limiting membrane [65].

Cell-to-cell communication is regulated by the mediators bound on the surface of sEVs or those found in the sEV lumen [71]. Then sEVs traverse to the extracellular space of the neighbouring cells, or passively transport through the bodily fluids or the bloodstream [66]. sEVs in circulation can even pass through the blood–brain barrier, hence also playing a significant role in distant cell-to-cell communication in biological processes [71]. sEVs carry multiple biological cargos including protein, nucleic acids and lipids, which are delivered among cells for paracrine and systemic cell-to-cell communication [69]. In particular, sEVs deliver small and large non-coding RNAs such as microRNA (miRNAs) that post-transcriptionally regulate gene expression [22, 72]. They also transport messenger RNAs (mRNAs) to recipient cells for protein synthesis [72]. For instance, mRNAs can be taken up by nearby or faraway cells when sEVs circulate, and eventually sEVs regulate the target cells [16]. Furthermore, sEVs can directly bind and activate cell surface receptors, including proteins and bioactive lipid ligands [20].

Immune and non-immune cells generate sEVs that modulate physiological processes, and for that reason may exhibit therapeutic potential in inflammatory and autoimmune diseases [72]. The innate and adaptive immunity, tissue repair, autophagy, stem cell maintenance, blood coagulation and angiogenesis are partially regulated via sEV release [69]. In immune cells, sEVs mediate immune stimulation by carrying signalling molecules and antigens, and once released, they interact with immune cells regulating their specific responses [20, 26]. For instance, sEVs can suppress inflammation through the transport of death ligands such as FasL and TRAIL thereby maintaining an immune tolerance [20]. Adaptive immune responses are also moderated by APC-derived sEVs through the presentation of antigens to the T cells thus influencing T cell activation and subsequent subset differentiation [20].

### T-sEVs secretion

Studies have shown that various types of cells have an identical pathway to sEVs release following MVE fusion with the plasma membrane [73, 74]. T cell activation appears essential to generate sEVs, and this involves complex processes facilitated by specialized proteins [61]. For instance, the T cell receptors (TCRs), integrins, lectins, transmembrane cytokines and cytokine receptors are present on the surface of T-sEVs [75]. The study by Li et al. found that T cells from the peripheral blood of healthy subjects released sEVs in response to T cell receptor stimulation [8]. Additionally, T-sEVs contain proteins that exert a role in immune surveillance, antigen recognition, immune activation and immune suppression [12]. For example, sEV can deliver major histocompatibility complex (MHC) class I and II molecules, T cell receptors, transmembrane cytokines such as TNF*α* or Fas ligand (FasL), as well as cytokine receptors, integrins, lectins, miRNAs and sphingolipids [12]. T-sEVs could also mediate the antiviral response by transporting mitochondrial DNA to dendritic cells [23]. Activated T cells display ability to secrete T-sEVs containing FasL, APO2 ligand and TCR/CD3 complexes, all of which are engaged in immune regulation through the stimulation of activation-induced T cell death (Table 1) [76].

**Table 1:** Roles of T-sEVs in immunomodulation

T cell subset	Composition of T-sEVs	Target cells	Mechanism of action/effect of T-sEVs	References
CD3^+^ T cell	n.a	T cells	Generated proliferation in resting T cells	[77]
CD3^+^ T cell	miRNA-335	APC	Transfer of miRNA from CD3^+^ T cell to APC	[86]
CD3^+^ T cell	FasL and APO2L	n.a	Mitogenic stimulation produced T-sEVs bearing FasL and APO2L	[87]
CD8^+^ T cell	Granzyme B and perforin	Mesenchymal tumour cells	Transport Granzyme B and perforin onto mesenchymal tumour cell, controlling tumour growth	[88, 89]
CD8^+^ T cell	Granzyme B and perforin	Melanoma animal models	Transport granzyme B and perforin onto melanoma, control of tumour growth	[90]
CD8^+^ T cell	FasL	Melanoma, lung cancer animal models	Transport of FasL causes invasion of melanoma tumour cells	[91]
CD8^+^ T cell	AXL-siRNA (siAXL) loaded glutathione (GSH)-responsive PTX-poly-L-lysine (PTX-PLL; PP) prodrug micelle	Breast cancer	Promotes PTX release to kill breast tumour cells	[92]
CD8^+^ T cell	miR186-5p	Kidney	miRNA mediates renal inflammation	[93]
CD8^+^ T cell	IL-12	Low affinity CD8^+^ T cells	T cell activation	[94]
CD8^+^ T cell	IL2 and cetuximab (engineered)	Lung cancer	Cytotoxicity	[95]
CD8^+^ T cell	IL-12	naïve CD8^+^ T cell	Granzyme B production	[81]
CD8^+^ T cell	TCR/CD3	n.a	n.a	[85]
CD8^+^ T cell	Cytotoxic granules	T helper cell	transfer of cytotoxic granules to target	[96]
CD8^+^ T cell	membrane-bound anti-HIV factor	HIV-1 infected CD4 T cells	Suppressed CCR5-tropic (R5) and CXCR4-tropic (X4) replication of HIV-1	[97]
CD8^+^ T cell	Perforin	n.a	Ensure unidirectional transport of Perforin to target cell	[98]
CD8^+^ T cell	FasL	n.a	T cell activation releases sEVs bearing FasL	[99]
CD8^+^ T cell	miR-765	Endometrial cancer	Suppression of tumour-promoting effects of estrogen on endometrial cancer	[100]
CD4^+^ T cell	Ovalbumin (model antigen)	Melanoma	Tumour progression	[76]
CD4^+^ T cell	miR-25-3p, miR155-5p, and miR-215-5p	Melanoma	Reduced tumour growth	[101]
CD4^+^ T cell	n.a	B cells	Increased activation and proliferation	[15]
CD8^+^ and CD4^+^ T cell	PD-L1	Breast cancer	Compete with tumour-PD-L1 binding to PD1 on T cells, restoring immune surveillance	[102]
CD8^+^ and CD4^+^ T cell	CD3 + HLA-DR +	n.a	Serum markers of in vivo T cell activation	[103]
CAR T cell	Granzyme B and perforin	Breast cancer	Suppression of tumour growth via granzyme B and perforin transfer	[104] [105]
Vδ2-T cells	FasL	Epstein-Barr virus (EBV)-transformed tumour cells	tumour cell killing	[106]
IL2-T cells	miR-181a-3p and miR-223-3p	Melanoma	Transport of miRNAs delayed tumour growth	[107]

Recent studies also indicated that myelin and lymphocyte (MAL) protein plays a role in T-sEV biogenesis [7]. MAL tetraspanin membrane protein (17 kDa) has been identified in the endoplasmic reticulum of T cells [7] and is responsible for carrying tyrosine kinase Lck to the T cell plasma membrane [65]. Once MAL is inhibited, the numbers of secreted T-sEVs are decreased, which is in agreement with the reduced sorting of CD63 into the intraluminal space of MVE, and its assembly on the limiting membrane of MVE [65]. Basically, the key role of MAL protein is the organization of condensed membrane domains for T-sEV biogenesis [65].

### T-sEVs composition and functions

T cell activation through TCR leads to secretion of T-sEVs (Fig. 1a) [77]. In particular, activation via TCR and CD28 leads to an increase in T-sEVs release [78, 79]. T-sEVs bear CD3 [75] and TCR and contain cytoplasmic/endosomal proteins such as tumour susceptibility gene TSG101 and heat shock protein Hsp70 [7], along with sphingomyelin and cholesterol as demonstrated by lipidomic analyses [65]. This is consistent with the assembly of a lipid-metabolizing enzyme diacylglycerol kinase α, on the limiting membrane of T cell MVB, that is responsible for MVB maturation and the secretion of CD63-bearing sEVs in T cells, as demonstrated by Ventimiglia and Alonso [65]. Remarkably, suppression of diacylglycerol kinase α led to activation-induced cell death in certain T cells, via augmented release of sEVs containing the lethal Fas ligand [80]. Overall, T-sEV proteins found on the surface or within human and mouse T cells, include Actin, Alix, CD2, CD3, CD4, CD25, CD47, CD63, CXCR4, FasL, Hsp90, LFA-1, MHC-I, MHC-II, TCR, TSG101, Tubulin and Lck [80–82].

T-sEVs are involved in modulation of immune responses, and as such T-sEVs have been explored for their potential as immunomodulators [83]. T-sEVs also engage in the transport of activating or suppressive factors to target cells, leading to activation of other immune cells, or inhibition of the immune response [28, 84]. T-sEVs released from activated T cells, can promote naïve T cell activation [78]. For example, IL2-containing T-sEVs could directly induce CD8^+^ T cell proliferation [78]. T-sEVs, released from activated T cells, were shown to activate naïve CD8^+^ T cells (in the presence of IL2), with IFNγ and Granzyme B production [78]. Blanchard et al. showed that T-sEVs secreted by a T cell line following activation, contained CD3/TCR complexes, and that activated T cells released more CD3/TCR-containing sEVs than resting T cells (Table 1) [85]. Such CD3/TCR-containing T-sEVs may be relevant for binding with MHC-peptide complexes on APCs or other targets. In addition, tetraspanin CD82 was involved in T cell activation by regulating the actin cytoskeleton, as demonstrated by Ventimiglia and colleagues [65]. In summary, CD3/TCR, adhesion molecules, CD82 and Src tyrosine kinases were shown to be associated with T-sEVs [65, 85]. Table 1 summarizes the key functions T-sEVs released from principal T cell subsets.

### T-sEVs in the intercellular communications

T-sEVs have been recognized as mediators of intercellular communication, capable of transporting specialized molecules to their target cells [108]. Several reports focussed on the immune synapse (IS) formed between the T cell and APC, which enables a distinct localization of the intercellular communication between T lymphocytes and MHC-containing APCs, enhances specificity, and is essential in T cell activation and transport of intercellular signals and effector molecules [64, 67]. Studies by Mittelbrunn and Hermann found that IS allows for a direct transfer of miRNA-containing sEVs [69, 86]. Thus, IS enables effectiveness of antigen presentation, including by miRNA transfer [109, 110].

*CD8*^+^ *T-sEVs.* T-sEVs act similar to their parent T cells, engaging with antigen-presenting cells, and destroying target cells [28]. On the other hand, T-sEVs may play a part in cancer progression, such as in metastasis, invasion and cancer-associated inflammatory response [69]. T-sEVs gained attention in cancer immunotherapy for their role as regulators of the anti-tumour–immune response [28, 30]. CD8^+^ T-sEVs carry effector cytokines TNFα and IFNγ, and cytotoxic granules containing perforin and Granzyme B (Fig. 1b) [111]. Granzymes are transported following fusion of CD8^+^ T-sEVs with the membrane of the target cells, or via the endocytic pathway [111, 112]. IL-12-induced CD8^+^ T-sEVs contained high levels of immunoregulatory molecules (Granzyme B, STAT3, STAT5) [85], and these T-sEVs could upregulate IFNγ and Granzyme B in naïve CD8^+^ T cells, in the absence of antigen recognition (Fig. 1b) [85].

**Fig. 2:**
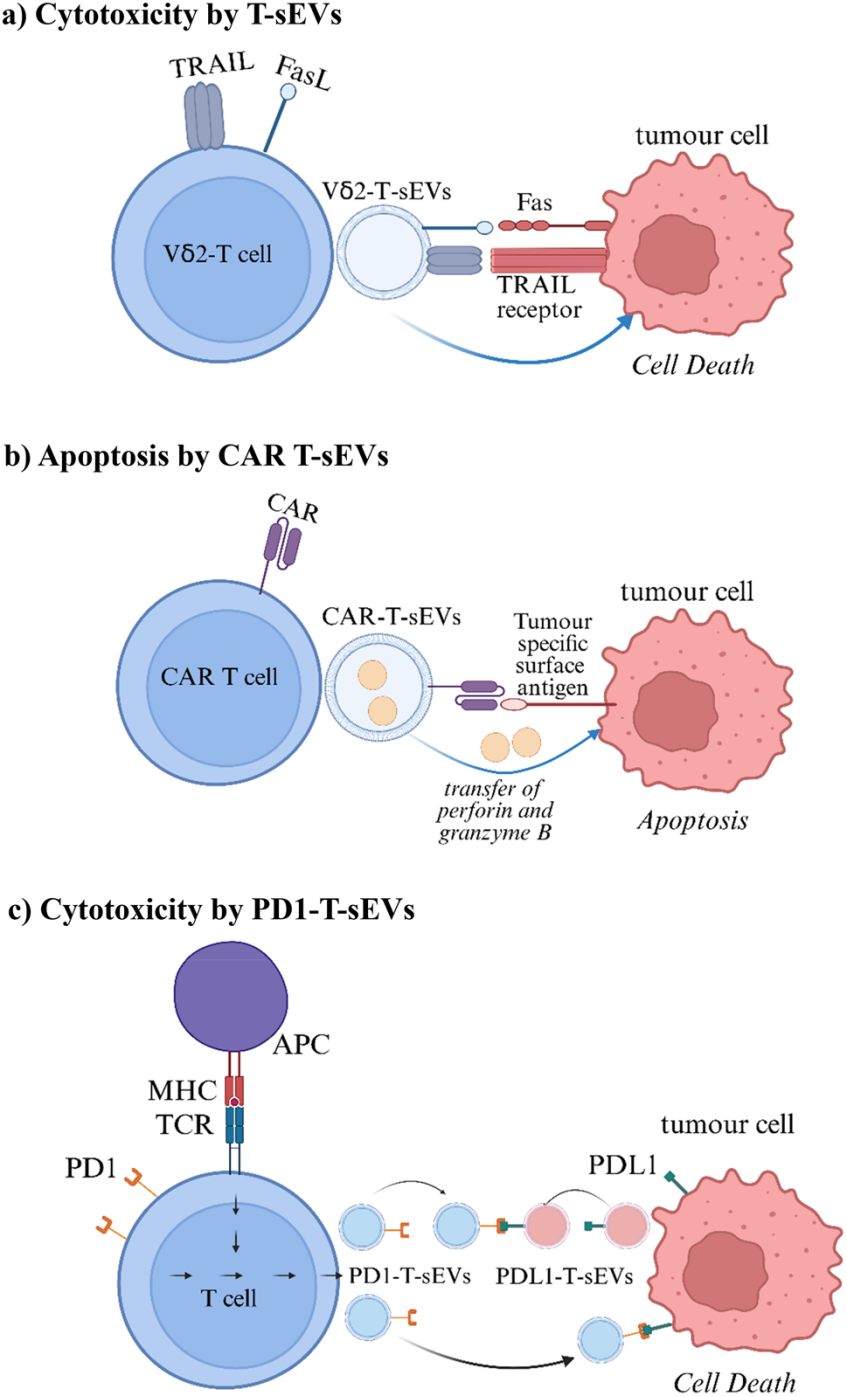
Anti-tumour effects of T-sEVs. **a** Vδ2-T-sEVs expressing FasL and TRAIL proteins displayed cytotoxicity against tumour cells (106). **b** CAR-T cells generated sEVs which carried CAR on their surface, and expressed Granzyme B and perforin which caused apoptosis of tumour cells (104). **c** PD1-expressing T-sEVs were found to block the PD1:PDL1 interaction thus mediating tumour cell death (102). (Created with BioRender.com)

Xie and colleagues demonstrated that T-sEVs from antigen-specific CD8 + T cells regulated immune responses through downregulation of MHC-I and dendritic cell killing, thus limiting further T cell stimulation [113]. Similarly, Borst et al. reported that T-sEVs released from antigen-specific CD8^+^ T cells suppressed a further CD8^+^ T cell response, reducing clonal competition—a finding suggesting immunosuppressive potential for the management of autoimmunity and organ transplant rejection [114]. Exhausted CD8^+^ T cells (a subtype of CD8^+^ T cells present at sites of chronic antigenic stimulation such as in tumours or sites of a chronic viral infection), liberated sEVs that were engulfed by non-exhausted CD8^+^ T cells, in turn reducing their potential to divide and kill targets [115, 116].

The cell surface protein Fas from the TNF family functions in inducing apoptosis in Fas-bearing cells after engaging with the cognate Fas ligand (FasL) [91]. T cells express FasL following T cell activation (Table 1). Li and colleagues observed that once T-sEVs derived from CD8^+^ T cells were modified to overexpress PD1, such T-sEVs were found to contain Granzyme and FasL (Table 1) [90]. Cai et al. showed that FasL-containing T-sEVs from activated T cells could enhance tumour invasion in vivo through the stimulation of Matrix metalloproteinase 9 (MMP9) [91]. In this model, CD8^+^ T cells and FasL T-sEV were present in tumour tissues, indicating that CD8^+^ T cells repeatedly released FasL T-sEV to facilitate tumour development instead of inhibiting it [91].

CD8 T-sEVs containing AXL-siRNA (siAXL) loaded glutathione (GSH)-responsive PTX-poly-L-lysine (PTX-PLL; PP) prodrug micelle were found to induce paclitaxel (PTX) release which then destroyed breast cancer cells [92]. PTX is a breast cancer chemotherapeutic agent whose function is to supress cell division through strengthening of the microtubules. AXL receptor tyrosine kinase has exhibited elevated levels in breast cancer and induces tumour growth. PTX together with poly-L-lysine (PLL) was mechanistically combined using glutathione-responsive disulfide bonds for the formation of PTX-SS-PLL which through self-assembly led to tumour-specific prodrug micelle development. siRNA was taken up on the micelle exterior via electrostatic interaction with PLL; then, these were absorbed by T-sEVs [92].

*CD4*^+^ *T-sEVs.* CD4^+^ T-sEVs play a role in helping adaptive immune responses [116]. CD4^+^ T-sEVs contain proteins such as TCR, LFA-1, CD4, CD25 and FasL (Fig. 1c) [116]. Kalluri and colleagues demonstrated that CD4^+^ T-sEVs serve as immunomodulators by augmenting Hepatitis B antibody (HBsAb) responses in HBsAg-vaccinated mice [21]. Following immunization with HBsAg vaccine, CD4^+^ T-sEVs contributed to enhanced humoral immune responses as illustrated by high levels of HBsAb in serum, and numbers of plasma cells in the bone marrow [21]; in vitro, pharmacological inhibition of sEV production reduced levels of IgG and decreased expression of MHC-II and CD86 on B cells (Table 1) [9]. Zhang et al. used flow cytometry to demonstrate that CD4^+^ T-sEVs contained TCR, CD25, FasL and LFA-1[76]. Moreover, T-sEVs derived from activated, antigen-specific CD4 T cells, potently suppressed anti-tumour cytotoxic responses in the animal model of melanoma [76]. As a whole, these findings illustrate that T-sEVs play a critical role in immune regulation [3].

Oba et al. enabled measurement of CD4^+^ T-sEVs, CD8^+^ T-sEVs and CD3^+^ MHC-II^+^ EVs in serum, as a measure of in vivo activation for CD4^+^ T, CD8^+^ T and Th1/Tc1-type T cells, respectively (Table 1) [103]. CD4^+^ and CD8^+^ T cells purified from the blood of healthy donors and stimulated with CD3/CD28 antibodies, generated significantly higher amounts of CD4^+^ T-sEV and CD8 + T-sEVs compared to the unstimulated cells [103]. Nagasawa et al. found higher amounts of CD4^+^ and CD8^+^ derived T-sEVs in serum from patients with acute graft-versus-host disease (GVHD), in agreement with widespread T cell activation in this condition [117].

Regulatory T cells (Tregs) are a distinct subset of CD4 T cells associated with immune suppression [118]. Treg-sEVs mediated peripheral tolerance by suppressing CD8^+^ and CD4^+^ T cell responses [118]. Okoye et al. demonstrated that Treg-derived T-sEV carried miRNAs and mediated suppression of Th1 cells [119]. Specifically, Let-7d miRNA contained in sEVs from Treg cells, inhibited T helper cell proliferation and IFNγ release [119].

## T-sEVs up-to-date innovations

### miRNA-containing T-sEVs

Comprehensive analysis of the composition and function of T-sEVs, along with insights from earlier studies, may help fill the critical knowledge gaps and guide the development of engineered T-sEV and their potential for application in preclinical cancer models. Fusion of T-sEVs with the plasma membrane of the recipient cells unloads the cargo of functional miRNA into their cytoplasm [120]. Seo et al. demonstrated that mouse T-sEVs could exert anti-tumour effects by targeting tumour stroma and eliminating tumour-associated mesenchymal stromal cells (Table 1) [89]. Mesenchymal stromal cells engulfed T-sEVs and underwent apoptosis that was dependent on miRNA (miR-298-5p) embedded in T-sEVs [89]. Destruction of mesenchymal stroma by CD8^+^ T-sEVs reduced invasion and metastasis of transplantable melanoma (B16F10), breast cancer (4T1) and sarcoma (CMS5m) tumours, in subcutaneous and metastatic mouse models [89]. Shin and colleagues indicated that sEVs released by CD4^+^ T cells, stimulated CD8 + T cell proliferation and cytotoxicity [101]. This was confirmed by improved CD8^+^ T cell killing of tumour cells in vitro and delayed growth of syngeneic B16 melanoma tumours in mice after intratumoural administration of CD4^+^ T cell-derived sEVs (Table 1) [101]. Mechanistically, the effect was mediated by miR-25-3p, miR-155-5p, miR-215-5p and miR-375 [101]. The potential risks in miRNA loading include: (i) uncertainty to their specificity to the disease, (ii) delivery of a specific miRNA is not a guarantee that the disease will be suppressed, (iii) a family of miRNA may be necessary to guarantee the downstream and upstream effects on the recipient cells [13].

T-sEV cargo loading could be manipulated through endogenous and exogenous approaches [121]. Endogenous cargo loading could be altered via parental cell modification, such as cellular bioengineering with plasmids and viral vectors [121]. Jung et al. bioengineered T cells to stably express membrane-bound IL2, and such cells produced IL2-containing sEVs [107]. The authors showed that IL2-sEVs decreased melanoma sEV production and melanoma-PD-L1 expression, mediated through miR-181a-3p and miR-223-3p (Table 1) [107]. Furthermore, treatment with IL2-T-sEVs synergized with chemotherapy and immunotherapy in B16F10 mouse melanoma model [107].

### Cytotoxic protein-expressing T-sEVs

Vδ2-T cells, a subset of gamma delta T cells, produced T-sEV enriched in cytotoxic molecules (Fas ligand, TRAIL), immunostimulatory ligands (C80/CD86) and antigen-presenting molecules (MHC class I and II) (Table 1 and Fig. 2a) [106]. Vδ2-T-sEVs were directly cytotoxic to EBV-associated tumours such as B cell lymphomas, and mediated complete control of such tumours in immunodeficient mice following systemic (intraperitoneal) administration, indicating a direct therapeutic potential for cell-free preparations of sEVs derived from gamma delta T cells [106]. On the other hand, chimeric antigen receptor (CAR) T cells genetically engineered to express tumour antigen-specific CAR are used to treat a range of B cell malignancies [104]. Fu and colleagues revealed that sEVs released by CAR-T cells (CAR-T-sEVs) carry multiple cytotoxic molecules (Granzyme B and perforin) and could suppress tumour growth in xenograft models (Table 1 and Fig. 2b), likely via direct tumour killing [104]. Meanwhile, there is a growth in expanding the applicability of allogeneic CAR-T cells through programmable nucleases such as short hairpin RNAs (shRNAs) for the knockdown of TCR or MHC to hinder the potential risk of immune rejection of allogeneic CAR-T cells [122].

Qiu and colleagues denoted that activated human T cells produced T-sEVs containing PD1, and that these PD1-T-sEVs had anti-tumour activity in vitro and in mouse models (Table 1) [102]. Mechanistically, PD1-sEVs interacted with cellular PD-L1, triggering PD-L1 internalization and therefore a reduction in PD-L1 expression on tumour cell surface (Fig. 2c). This in turn enhanced T cell cytotoxicity against tumour cells by preventing PD1 mediated T cell dysfunction [102]. On the other hand, Li et al. demonstrated that sEVs overexpressing PD1 (sEV-PD1) had direct and indirect anti-tumour activity in mouse melanoma model (in vitro and in vivo) (Fig. 2d) [90]. When injected intravenously into mice, sEV-PD1 inhibited the growth of B16F10 tumours in subcutaneous and lung metastasis models [90].

Additionally, the mechanism of the regulation of sEV biodistribution and clearance is also very important [20]. The in vivo zebra fish embryo-derived sEV biodistribution can be evaluated using single photon emission computed tomography (SPECT), bioluminescence, immunofluorescence and fluorescent labelling of proteins and lipids to name a few [20, 123]. However, these are unable to trace the sEV, and the solution is using approaches displaying massive temporal and spatial resolution [20].

CRISPR technology is a gene-editing tool applied in cancer cell-derived sEVs and is also used in cancer therapeutics [124]. A current approach is EXTRA-CRISPR (Endonucleolytically eXponenTiated Rolling circle Amplification with CRISPR–Cas12a); this is similar to RT-qPCR in terms of sEV marker detection and is a labour-saving and straightforward [124].

## Perspectives and future directions

T-sEVs are emerging as versatile players in a variety of immune responses, as mediators in intercellular communications through immunomodulation or direct killing of target cells via release of cytotoxic cargo [9]. sEVs carrying molecular signatures of their parental cells can be recovered from a variety of biological fluids, highlighting sEV potential as non-invasive biomarkers in preclinical and clinical research [9]. Liquid biopsies capturing T-sEV numbers and signatures may allow for monitoring of immune responses in cancer patient receiving a range of immunotherapies, from immune checkpoint blockade to adoptive cell therapies including CAR-T cell-based therapies [9, 104]. On the other hand, further advances in ex vivo T-sEV bioengineering aimed at increasing T-sEV anti-tumour properties may add to the arsenal of new immunotherapeutics available for cancer treatment [9]. Yet there are several challenges to overcome before T-sEVs can be used in cancer diagnostics or cancer treatment.

At present, there is insufficient clinical validation data on T-sEV applications. For example, reproducible PCR validation of the genes classified by sEV-RNA sequencing is a common approach [125]. However, RT-qPCR validation is critically dependent on the presence of the entire target region which sEVs may not contain. Thus, it is not suggested to use PCR primer design by cumulative total expression of a specified gene. It is advised to design PCR primers through conservative recognition of the genomic region (genetic loci) of RNA which demonstrates the elevated sEV expression [125].

PD-L1 expression on tumour-derived sEVs has been proposed as a predictor of clinical responses to anti-PD1 therapy in melanoma [126]. PD-L1 expression on tumour cells or in the tumour microenvironment is increasingly used as a predictive biomarker of immunotherapy response in solid tumours. However, pathological evaluation of PD-L1 expression in solid tumours is complex, with different methods of assessment in different tumour types impacting on results and hence on therapeutic decision-making [126]. PD-L1 associated with sEVs may provide an additional evaluation method, contributing to enhanced prognostication**.**

miRNAs play a role in regulating gene expression as such they also function in immune modulation [125]. In hepatocellular carcinoma, sEVs-miRNAs levels were elevated compared to healthy patients, indicating potential role as a prognostic biomarker [125]. Long non-coding RNAs (lncRNAs) are emerging diagnostic biomarkers, since increased levels of specific lncRNAs were present in circulating sEVs in cervical, liver and breast cancer patients [125].

In addition, miRNA-containing sEVs from tumour cells were found to be elevated in comparison with healthy patients, implying the high potential of various miRNAs as hepatocellular carcinoma diagnostic and prognostic biomarkers [125]. It is advised to apply standardized detection procedures in clinical applications to avoid biased outcomes in sEV-RNA detection [125]. Moreover, standardized bioinformatics pipelines, comprehensive transcriptomic assessment not only in miRNAs, reference agnostic approaches and reference mapping are also recommended [125].

Despite the technological progress in T-sEV isolation, standard isolation methods such as ultracentrifugation, size exclusion chromatography (SEC) or antibody-based capture remain labour intensive, time-consuming and currently, impractical for real-time clinical application [127]. Commonly copurified non-lipid particles such as protein aggregates, and lipid particles-like substances such as micelles, as well as low yields of T-sEVs isolated from serum or plasma, limit the potential diagnostic utility of the purified products [127]. Furthermore, microfluidics-based isolation methods may help improve T-sEV purity but currently do not address T-sEV cell subset derivation (CD4^+^ versus CD8^+^ T cells, or functional subsets such as effector memory, Th1 or Treg cells) [128]. As described in section "T-sEVs in the intercellular communications", sEVs, derived from CD4^+^ and CD8^+^ T cells, have distinct functions—hence precise and reliable methods to isolate specific phenotypes need to be incorporated in microfluidic isolation systems. To achieve this, further studies are required to delineate the complexity of T-sEVs derived from various T cell subsets, and capture the molecular signatures that could be used in microfluidics T-sEV isolation devices [128].

Furthermore, the rate of release and composition of T-sEVs can be modified by local cues such as those in the tumour microenvironment [9]. These effects can be direct, such as the composition of tumour-infiltrating lymphocytes (cytotoxic versus regulatory), and indirect such as modulation of the rate of T-sEV release through T cell functional states (activated versus exhausted T cells) and presence of myeloid immunomodulatory subsets such as tumour-associated macrophages, dendritic cells and myeloid-derived suppressor cells. Furthermore, the initial T-sEV trafficking via the lymphatics and/or blood, and the rate of sentinel lymph node retention, will affect plasma T-sEV concentrations and hence their recovery [9].

Tumour-associated macrophages (TAMs) and myeloid-derived suppressor cells (MDSCs) are known to promote tumour growth through various means, including by inactivating tumour-reactive T cells. Importantly, TAMs express high levels of antigen-presenting molecules which, coupled with their ability to acquire tumour-derived antigens within the microenvironment, makes TAMs important antigen-presenting cells in situ. TAM-derived sEVs were found to carry high levels of the inhibitory ligand PD-L1, which could contribute to CD8 T cell inactivation directly by engaging PD1 on T cells and indirectly, by impacting the transcription factor PPARα causing T cell apoptosis via increased fatty acid oxidation and production of reactive oxygen species [129, 130]. In addition, TAM-sEVs may directly affect T cell trafficking into tumours, as siRNA targeting of TAM-specific Rab27a increased T cell infiltration and improved efficacy of anti-PD1 immunotherapy in the animal model of melanoma [129]. Systemic administration of MDSC-derived sEVs to healthy mice depleted circulating CD8 T cells while promoting M2 macrophage polarization and re-enforcing differentiation of immunosuppressive granulocytic MDSC [131]**.** MDSC-derived sEVs carried Fas ligand and promoted CD8 T cell apoptosis via Fas engagement and increased production of reactive oxygen species [131]. In turn, T-sEVs can be engineered to restrain MDSC development. Johnson et al. modified CAR-T cells to deliver RN7SL1, an endogenous RNA that activates type I interferon signalling [132]. CAR-sEVs carrying RN7SL1, restricted MDSC development, decreased immunosuppressive TGFβ in myeloid cells and promoted dendritic cell (DC) subsets with costimulatory features [132]**.**

Studies have demonstrated that hypoxia, which is characterized by a reduction in oxygen levels, elevates cancer cell-derived sEV activity and yields through prompting secretory lysosome phenotype [133, 134]. Hypoxia has an impact on sEV production by tumour cells and stromal elements such as TAMs and MDSCs [135]. Hypoxic epithelial ovarian cancer cells utilize macrophages and modify them into TAMs [135]. TAM-derived hypoxic sEVs transport miR-223 to ovarian cancer cells, presenting resistance to cisplatin via the PTEN/PI3K/AKT pathway [135]. While MDSCs influence the activated state of the T cell and sEV secretion through hypoxia, specifically hypoxia-inducible factor 1 α (HIF1α)-dependent manner [136]. Additionally, sEVs from hypoxic glioma cells enable the expansion of MDSC through miRNA-29a/Hbp1, miRNA-10a/Rora, miRNA-92a/Prkar1a and miRNA-21/PTEN pathways [137, 138]. Moreover, hypoxia present complex effects on TILs, it reduces T cell infiltration into tumours and drives the state of T cell exhaustion via various mechanisms such as glucose deprivation, accumulation of lactate and immunosuppressive adenosine produced via upregulation of CD39 on both T cells and macrophages on the TME [139–141]. However, the effect of hypoxia on T-sEV release is still poorly defined. Assuming that tumours display heterogeneity in hypoxic area distribution, with highly hypoxic areas consisting low number of T cells, the final effect on T-sEVs in systemic circulation is expected to be complex.

Aside from hypoxia, low pH can also influence sEV biogenesis [142]. Ban et al*.* have reported a study that an acidic environment upregulated the levels of sEV markers CD9 and CD63 following an in vitro analysis of cancer cell-derived sEVs [142]. Moreover, they also found that sEV proteins and RNA concentration were increased in acidic medium in comparison with neutral medium, indicating that an acidic environment is beneficial for sEV yield [142]. Furthermore, elevated stiffness of the extracellular matrix (ECM) stimulated the sEV secretion from tumour cells [143]. Alongside this, tumour cell-derived hypoxic sEVs carry out an immunosuppressive role by controlling the activity and function of immune cells [143]. All in all, most studies have showed the impact of physicochemical factors on the biogenesis of cancer cell-derived sEVs, little is known about T-sEVs. Our hypothesis is that T-sEVs exhibit more complex mechanisms and thus require further studies for substantial analysis.

Preclinical studies of targeted immunotherapies with bioengineered T-sEVs have largely used cargo modification (cytotoxic proteins or miRNA loading) and/or tailoring of membrane protein sequences to enhance T cell recognition and activation, in various ex vivo systems [89]. The knowledge is still limited, and further insights are required into: (i) T-sEVs biogenesis in response to various immune stimuli triggering T cell activation, and the precise window of T-sEV release, (ii) T-sEV content specificity with regards to proteins and nucleic acid composition and (iii) the precise effects of tumour microenvironment on T-sEV release. With future research likely focussing on the new approaches in T-sEV bioengineering, a deeper understanding of the complexity of T-sEVs machinery will be critical to ensure the robustness of the engineered T-sEV products, and the reproducibility of results across the world.

The heterogeneity of sEVs is impacted by size variation, sEV dynamic source, origin (normal or cancer cells) and molecular diversity [144]. Single sEV profiling is an integration of multi-omics (proteomics, genomics and transcriptomics) and AI (for data analysis) by linking clinical data analysis. sEVs and nanotechnology target isolation issues in a targeted manner for cancer theranostics, and it can also separate sEVs based on their molecular expression. Lastly, single sEV profiling is efficient in cancer biomarkers and appropriate therapeutic sEV molecular profiling for improved selection of proper sources of therapeutic sEVs [144, 145].

sEV barcoding is also applied in cancer diagnostics, monitoring and prognostics. This approach is used for accurate and distinctive sEV detection, thereby aiding in an in-depth understanding of diseases for optimal treatment. One type of sEV barcoding is chemical barcoding, wherein aptamers serve as sEV surface identifiers for precise sEV detection [144, 145]. Afterwards, they are analysed using sequencing techniques to provide a detailed sEV description. Using isolation methods, tagged sEVs are then extracted from bodily fluids. Another type is genetic barcoding; this involves engineering cells to yield sEVs containing DNA sequences which serves as a sEV label [144, 145]. With these developments, challenges such as heterogeneity will be addressed and will pave the way for novel techniques in personalized treatment and modernization in healthcare.

## Conclusions

In summary, T-sEVs are emerging as key players in immune responses. This review provides an extensive analysis of T-sEVs biology and their biological roles, and highlights current developments in T-sEV-based therapeutics. Precise understanding of T-sEV biology is critical for the development of T-sEV-based diagnostics and further optimisation of T-sEV bioengineered therapeutics. Evidently, the field is still in the exploratory stage; overcoming technical challenges of T-sEV isolation, while navigating the complexity of tumour–immune interactions accounting for the immune subsets, T cell activation states, duration of the response, local microenvironmental cues and the direct impact of tumour cells, create numerous difficulties for developing robust T-sEV-based diagnostics and therapeutics.

The T-sEV biological insights from preclinical cancer models are yet to be applied to clinical trials, and the great expectations for sEV-based therapeutics are yet to be fulfilled. Nevertheless, we believe that joint efforts from scientists, researchers and clinicians will result in the development of robust T-sEV-based diagnostics, and improved T-sEV bioengineering processes towards improved immunotherapies for cancer.

## Data Availability

No datasets were generated or analysed during the current study.
