# Correction: T cell-derived small extracellular vesicles in cancer–immune interactions

**DOI:** 10.1007/s00262-025-04170-5

**Published:** 2025-10-15

**Authors:** Ma Janelle Chichoco Garcia, Su Su Thae Hnit, Elena Shklovskaya, Yuling Wang

**Affiliations:** 1 School of Natural Sciences, Faculty of Science and Engineering, Macquarie University, Sydney, NSW 2109 Australia; 2 Macquarie Medical School, Faculty of Medicine, Health and Human Sciences, Macquarie University, Sydney, NSW 2109 Australia

**Correction to: Cancer Immunology, Immunotherapy (2025) 74:252** 10.1007/s00262-025-04109-w

In the original version of this article, the wrong figures appeared as Fig. 1 and Fig. 2. The Figs. 1 and 2 should have appeared as shown below.

**Fig. 1:**
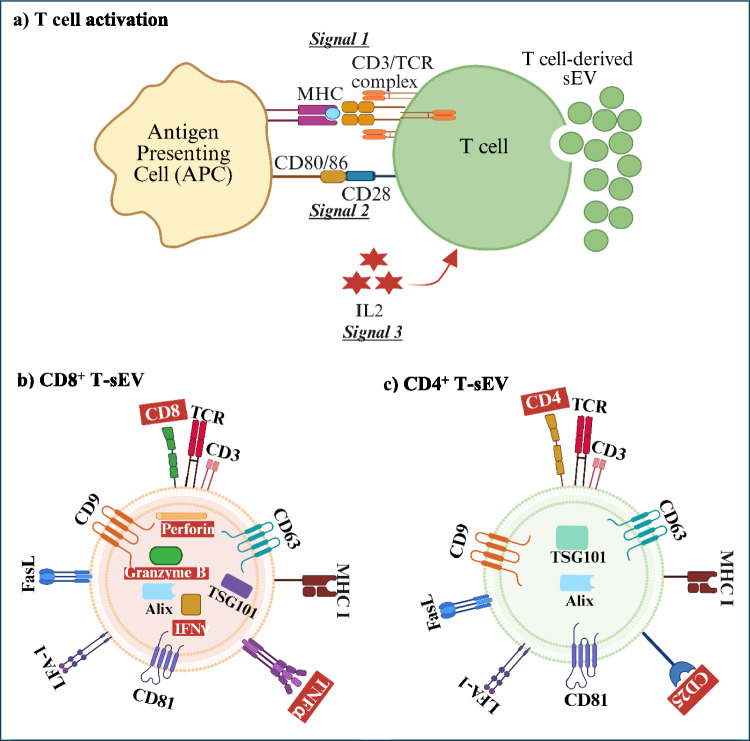
T-sEV generation by T cells activation and specific proteins found on CD8^+^ and CD4^+^ T-sEVs. **a** T cell activation is via Signal 1 which is provided by the TCR recognition of a peptide antigen bound to the MHC molecule on the APC. The second co-stimulatory signal (Signal 2) is provided by CD28 on T cells binding to CD80 and CD86 on APCs. The third signal (Signal 3) is T-cell derived IL2. The three signals integrate to stimulate sEV release from the T cell (32). **b** CD8^+^ T-sEVs and c) CD4^+^ T-sEVs both express the TCR/CD3 complex, MHC I, LFA-1, FasL and the sEV markers Alix, TSG101, CD9, CD63 and CD81. The key differences between CD8^+^ T-sEVs and CD4^+^ T-sEVs are highlighted in a red/dash box. CD8^+^ T-sEVs express TNFα, IFNγ, perforin and granzyme B (85). CD4.^+^ T-sEVs express CD25 (116). (Created with BioRender.com)

**Fig. 2:**
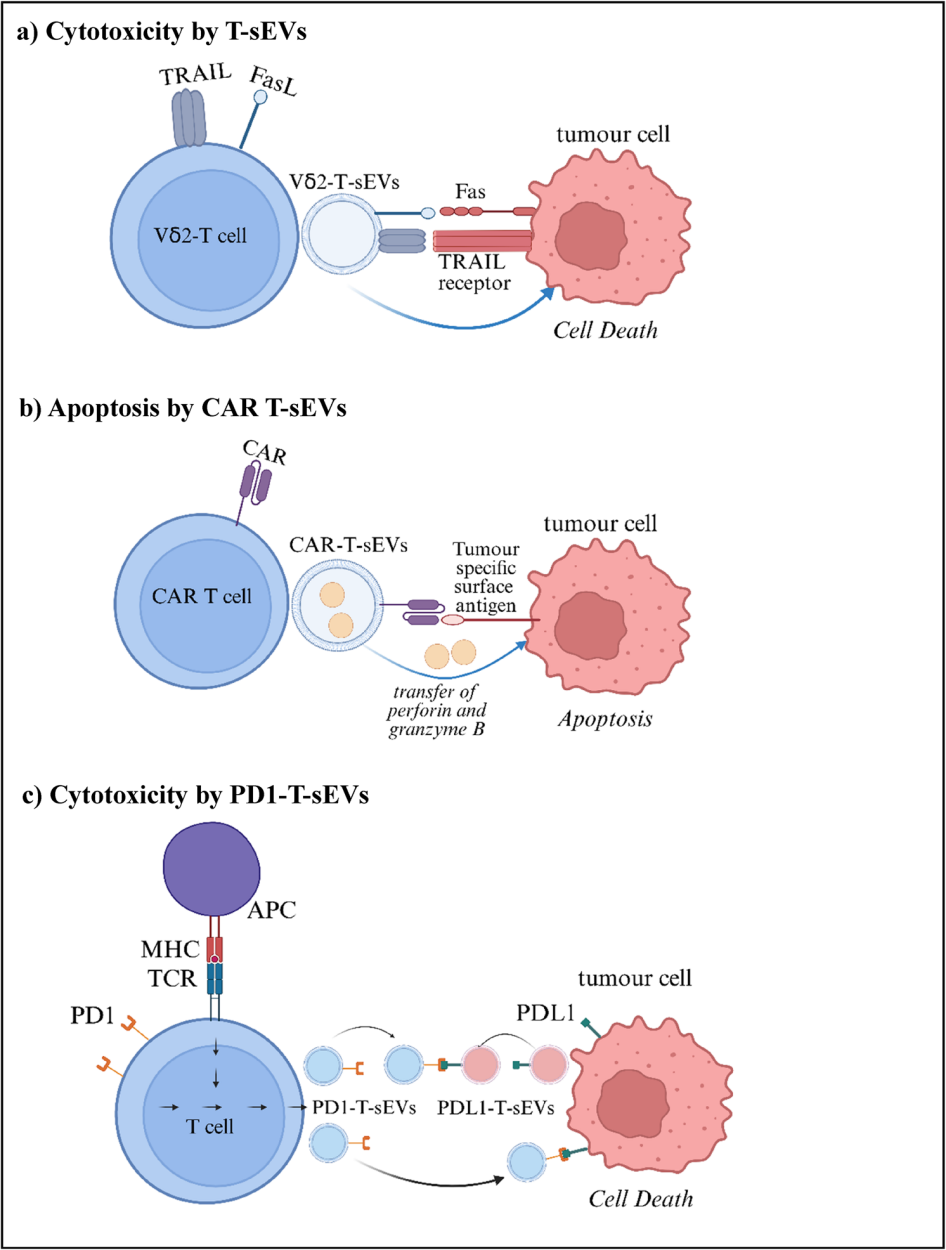
Anti-tumour effects of T-sEVs. **a** Vδ2-T-sEVs expressing FasL and TRAIL proteins displayed cytotoxicity against tumour cells (106). **b** CAR-T cells generated sEVs which carried CAR on their surface, and expressed Granzyme B and perforin which caused apoptosis of tumour cells (104). c) PD1-expressing T-sEVs were found to block the PD1:PD-L1 interaction thus mediating tumour cell death (102). (Created with BioRender.com)

The original article has been corrected.

